# Pedigree-based analyses of changes in genetic variability in three major swine breeds in Taiwan after a disease outbreak

**DOI:** 10.1093/tas/txac043

**Published:** 2022-04-13

**Authors:** Ruei-Syuan Wu, Hsu-Chang Wang, Chan Liang Su, Pei-Hwa Wang, En-Chung Lin

**Affiliations:** 1 Department of Animal Science and Technology, National Taiwan University, 10617 Taipei, Taiwan; 2 National Animal Industry Foundation, 10070 Taipei, Taiwan; 3 Dairy Association of Taiwan, R. O. C., 10644 Taipei, Taiwan

**Keywords:** genetic diversity, pedigree, random genetic drift, swine

## Abstract

Pedigree analysis was performed in three major Taiwanese swine breeds to evaluate the genetic variability in the current population and determine the main reason for genetic diversity (GD) loss after the occurrence of foot-and-mouth disease (FMD) in Taiwan. The pedigree files of the Duroc, Landrace, and Yorkshire breeds, containing 60,237, 87,177, and 34,373 records, respectively, were analyzed. We divided the population into two subpopulations (pre-1998 and post-1998) to determine the role of FMD in GD loss. Pedigree completeness and related indicators were analyzed to evaluate the pedigree quality, and several parameters were used to measure the levels of GD and further used to determine the major cause of GD loss. The pedigree completeness indexes for the different breeds were higher than 0.60, and the trend was enhanced after the FMD outbreak. The estimated proportion of random genetic drift in GD loss increased in all breeds over time (from 62.64% to 78.44% in Duroc; from 26.26% to 57.99% in Landrace; and from 47.97% to 55.00% in Yorkshire, respectively). The effective population size of Duroc and Landrace were increased by the time (Duroc: from 61.73 to 84.75; Landrace: from 108.70 to 113.64); however, it shows opposite trend in Yorkshire population (decline from 86.21 to 50.00). In summary, the occurrence of FMD led to the major loss of GD loss by random genetic drift. Therefore, for the recovery of GD, breeders in Taiwan should increase the effective population size with newly imported genetic materials and adjust the breeding strategy to reduce the inbreeding rate.

## INTRODUCTION

Genetic variability is used to represent the different genetic characteristics within or between breeds. In livestock, breeders typically use within-breed genetic variability as an indicator for allowing sustained genetic improvement for economically important traits to enhance performance (Reist-Marti et al., 2003), and it is helpful to make breeding objectives to meet particular market (Notter, 1999). However, due to the modification of production systems and breeding strategy, genetic diversity (GD) has been lost in swine breeds (FAO, 2000; Barker, 2001). The GD, or the expected heterozygosity, is a basic criterion for evaluating the genetic variability (Nei, 1973). The reduction of GD is associated with various events, such as inbreeding, which is usually associated with a negative influence on fitness-related traits (Fernández et al., 2005) and limit the response of selection (Falconer and Mackay, 1996). In closed populations, inbreeding leads to a higher rate of loss of alleles (Woolliams, 2007) due to unequal founder contribution. In contrast, even without inbreeding, the frequency of alleles in a certain generation may randomly increase, decrease, or even disappear in the subsequent generation. Genetic drift can eliminate alleles, and the disappearance of such genetic variation can only be restored by mutation or migration (Lacy, 1989). Therefore, understanding the genetic variability within a breed is necessary for confirming the value of genetic resources for future improvement and application. For instance, other countries have built up registry databases to collect comprehensive information for evaluating the genetic variability of their national purebred swine populations (e.g., USA: National Swine Registry and Canada: Canadian Centre for Swine Improvement).

**Figure 1.**
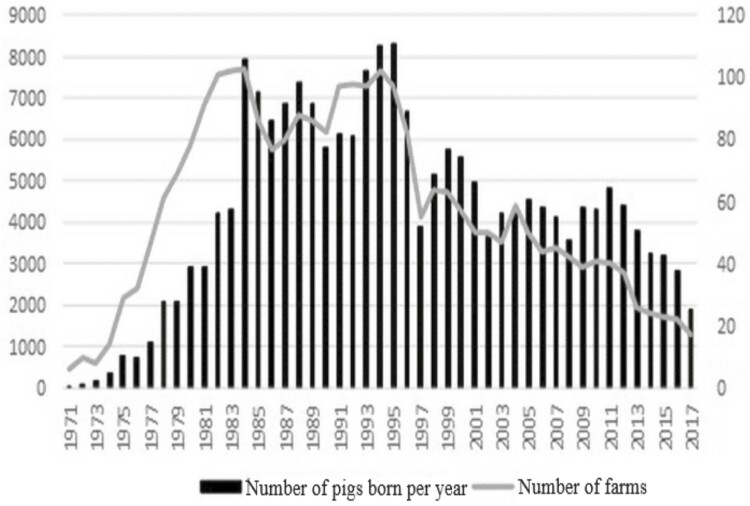
Number of pigs born and farm per year.

A purebred swine registry system was also established in Taiwan in 1975, beginning with a primary focus on recording superior imported livestock and continuing to collect records of their mating, farrowing, progeny, and performance testing to construct a complete pedigree with performance information. This information is useful for breeders (and some producers) to select or cull of their breeding stocks. At the same time, tracking the selection strategy based on pedigree and performance information has also strengthened the functionality of the registry system (Sung, 1993). The registration of purebred herds has become routine in Taiwan today, and until 2017, there had been more than 200,000 registered pigs (Fig. 1). However, there still has not been an in-depth study of the within-breed genetic variability of the Taiwanese purebred swine population. Moreover, in 1997, there was an outbreak of foot-and-mouth disease (FMD) in Taiwan, which resulted in the culling of around 2.73 million pigs (25.5% of the entire number of heads raised) that year. Noticeably, this cull included about 470,000 purebred animals (31.9% of the purebred herd) (COA, 1997). Although breeders have continued to import new genetics since 1998, the total number of purebred animals in the last two decades are only one-fifth of that before 1998. Rapid and persistent declines in population size are important causes of inbreeding and loss of genetic variability (Nei et al., 1975). Therefore, this study analyzes the variation in the GD of the swine populations before and after 1998 to understand the impact of FMD occurrence on the GD of purebred stocks in Taiwan. This study uses pedigree data to present the current GD of the three major pig breeds in Taiwan and identify the reasons for the loss of GD.

## MATERIALS AND METHODS

Animal Care and Use Committee approval was not obtained for this study because the study did not utilize any live animals.

### Characterization of the Pedigree Information

**Figure 2.**
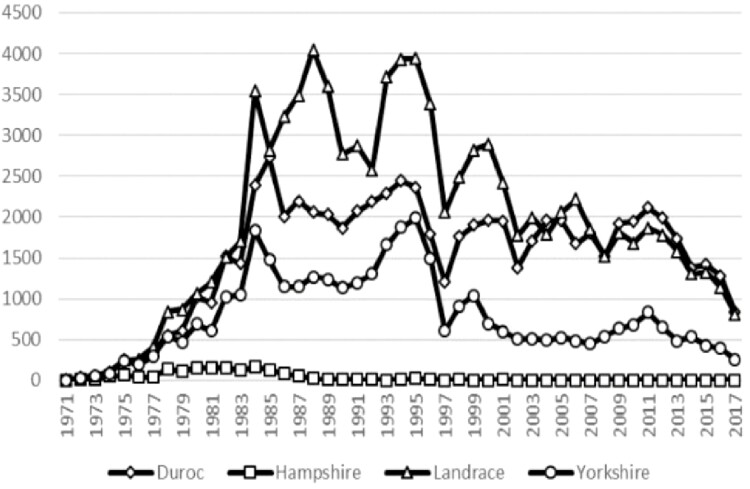
Number of pigs born within different swine breeds per year.

There are four imported breeds (Duroc, Hampshire, Landrace, and Yorkshire) that have been used by breeders in the crossbreeding systems in Taiwan. Duroc and Hampshire are the primary paternal breeds, and Landrace and Yorkshire are mainly used to produce the commercial parental sow (PS) for future commercial herd production. Due to Hampshire-crosses have a higher incidence of pale soft exudative (PSE) compared to Duroc-crosses (Purchas et al., 1990), which in turn leads to major economic losses for breeders, breeders in Taiwan gradually select Duroc breed as terminal sire breed over the Hampshire breed. Therefore, the registered number of Hampshire swine is significantly fewer than the other three breeds historically (Fig. 2). Thus, in this study, the analyses of genetic variability will focus on the Duroc, Landrace, and Yorkshire breeds. Pedigree records were obtained from the local database maintained by the National Animal Industry Foundation and included all pedigree records available for each breed up to 2017. To understand the influence of FMD disease on the genetic variation of purebred pigs, the entire population was divided into two parts: the subpopulation pre-1998, which includes pigs born between 1971 and 1997, and the subpopulation post-1998, which contains pigs born from 1998 to 2017. For comparison of GD, some population with GD management was often selected as a reference point. This is called the reference population, which can be some population in the past or the current population (Meuwissen et al., 2020). Previous literature has pointed out that average generation interval (GI) is used for reference population selection, which due to this reference population would comprises the last generation of data evaluated in each breed (Melka and Schenkel, 2010). Similarly, present study also used average GI as the basis for the selection of the reference population. Since the average GI of each breed in Taiwan is about 2.5 years, piglets born in 1995–1997 and 2015–2017 were defined as reference population for the pre-1998 and post-1998 subpopulation, respectively. The completeness of the pedigrees and the genetic variability were found by using pedigree information to calculate the associated parameters, such as pedigree completeness index, inbreeding coefficient and the parameters derived from probability of gene origin, etc. The number of animals analyzed in the entire pedigree and the reference population of each breed is given in Table 1. Pedigree files collected for the Duroc, Landrace, and Yorkshire breeds consisted of 60,237, 87,177, and 34,373 records, respectively, for subsequent analyses.

**Table 1.** Basic description of pedigree datasets, and the parameters of pedigree completeness and inbreeding analysis for all breeds.

Breed	Duroc		Landrace		Yorkshire	
subpopulation	Pre-1998	Post-1998	Pre-1998	Post-1998	Pre-1998	Post-1998
No. of animals in whole population	32,858	27,379	51,263	35,914	22,964	11,409
No. of animals in reference population	4,534	2,871	8,766	3,402	3,688	1,083
No. of Inbred animals	3,367	2,623	3,657	2,278	1,531	666
Inbred animals, %	74.26	91.36	41.71	66.96	41.51	61.49
Pedigree Completeness Index, %	0.83	0.86	0.64	0.73	0.61	0.69
Maximum generations traced	17	17	15	16	16	15
Mean equivalent generations	3.50	3.46	2.43	2.63	2.36	2.23
Complete generations	7	7	5	5	5	5
% known ancestors in:						
1st generation	92.9	96.7	92.0	91.3	90.7	88.1
2nd generation	85.6	91.0	72.5	82.2	70.7	77.4
4th generation	73.5	74.8	43.6	54.8	34.4	49.3
Mean *F*, %	4.24	3.62	1.37	1.76	1.86	2.77
Δ*F*, %	0.81	0.59	0.45	0.43	0.58	1.00
*N*_*e*_	61.73	84.75	108.70	113.64	86.21	50.00

Inbred animals, the animals with *F* > 0; Mean *F*, average inbreeding coefficient of all animals in reference population.

Δ*F*, the increase in inbreeding of all animals in reference population; *N*_*e*_, effective population size.

### Pedigree Quality Determination and Genetic Variability Evaluation

The ENDOG program (version 4.8) was used to identify the pedigree quality and genetic variability of these three purebred breeds (Gutiérrez and Goyache, 2005).

#### Pedigree Completeness

The pedigree completeness levels of the two reference populations were evaluated by calculating (1) the number of fully traced generations, (2) the maximum number of generations traced, and (3) the equivalent number of complete generations for each animal in the pedigree data. The first is defined as the number of generations that separates the offspring from the furthest generation where their ancestors of the individual are known. The second is the number of generations between an individual and its furthest ancestor. Finally, the equivalent complete generation is computed as the sum over all known ancestors of the terms computed as the sum of (1/2)^*n*^, where *n* is the number of generations separating the individual from each known ancestor (Maignel et al., 1996). Additionally, ENDOG offers a fourth indicator (4), the pedigree completeness index (PCI), which describes the completeness of each ancestor in the pedigree of the parental generation (MacCluer et al., 1983).

#### Generation Interval

The GI was computed for the four possible selection pathways (sire–son, sire–daughter, dam–son, and dam–daughter) as the average age of the parents at the birth of the offspring used to replace them.

### The Parameters for Genetic Variability Evaluation

#### Inbreeding and effective population size 

The individual inbreeding coefficient (F), which is defined as the probability that an individual has two identical genes by descent (Wright, 1922), was calculated according to the algorithm described by Meuwissen and Luo (1992). The increase in inbreeding (∆*F*) was calculated for each generation using the classical formula, Δ*F* = *F*_*t—*_*F*_*t-*1_/1 − *F*_*t*-1_, where *F*_*t*_ and *F*_*t*-1_ are the average inbreeding at the *t*th generation. The effective population size (*Ne*) represents the relationships between the number of males and females that contribute genetically to the population. Due to the increase of inbreeding, this parameter was estimated in this study (Wright, 1931) based on ∆ *F*, as *Ne = *1/(2△*F*), for each generation having *F*_*t*_ > *F*_*t*-1_, to roughly characterize the effects of remote and close inbreeding. *Ne* is defined as the number of breeding animals that would increase inbreeding if they contributed equally to the next generation (Gutiérrez and Goyache, 2005).

#### The probability of gene origin.

Total number of founders (*f*) was defined as ancestors with unknown parents. The effective number of founders (*f*_*e*_) means the number of equally contributing founders that would be expected to generate the same amount of GD as in the studied population (Lacy, 1989), was calculated using the following formula:


$$\begin{array}{l} f_{e}={\left[\underset{i=1}{\overset{f}{\sum }}\;q_{i}^{2}\right]}^{-1} \end{array}$$

where *q*_*i*_ is the genetic contribution of the *i*th founder to the reference population and *f* is the total number of founders. The *f*_*e*_ is usually lower than f, indicating the unequal contributions of founders due to selection, which is the major mating operation in the purebred industry. However, *f*_*e*_ alone may not be an useful indicator for assessing GD because the genetic contributions of the founders would converge after some number of generations (Bijma and Woolliams, 1999), and hence, the *f*_*e*_ would remain constant in the resulting population over time. The founder genome equivalent was defined as the number of equally contributing founders with no random loss of founder alleles that would be expected to give the same level of GD observed in the population under study (Lacy, 1989), and it was computed as *f*_*ge*_ = 1/(2 × *f*_*g*_), where *f*_*g*_ is the average co-ancestry for the population considered (Caballero and Toro, 2000). The amount of GD in the reference population was calculated as (Lacy, 1995):


$$\begin{array}{l} GD=1-\frac{1}{2fge} \end{array}$$

The loss of GD can be due to both genetic drift and unequal founder contribution; the value expressed as 1 − GD indicates the amount of GD lost in the population since the founder generation due to genetic drift and the unequal founder contribution. The amount of GD in the reference population accounting for the loss of diversity due to unequal founder contribution (GD*) was calculated as (Lacy, 1995):


$$\begin{array}{l} GD^{*}=1-\frac{1}{2f_{e}} \end{array}$$

1—GD* represents the loss of GD due to unequal contributions by the founders to the population (Caballero and Toro, 2000). Therefore, the difference between GD* and GD estimates the loss of diversity by genetic drift (Caballero and Toro, 2000; Honda et al., 2004).

## RESULTS

The number of purebred pigs began to grow significantly in 1980 and remained stable before the outbreak of FMD in Taiwan in 1997 (Fig. 1). However, in addition to the FMD outbreak in 1997, entry to the World Trade Organization (WTO) in 2002 and a worldwide rise in grain prices in 2008 caused a rapid decrease in the total number of pigs and breeders. Compared with the historical peak, the number of pigs born and the number of breeding farms have decreased by 66% and 76%, respectively, in the last 3 years. The international situation and the influence of breeding conditions have caused Taiwan’s pig breeding industry to become conservative and gradually formed a large-scale, centralized, and corporate feeding operation.

### Pedigree Completeness

The PCIs of the reference populations of different breeds are shown in Table 1. Over time, the PCI of the three breeds has increased. Comparison between the breeds indicated that the PCI of Duroc is the highest, while the completeness of the Yorkshire pedigree is relatively low. Similarly, the maximum generations traced and mean equivalent generations of the Duroc breed are also higher than the other two breeds. The analysis of known ancestors in different generations shows that in the first generation, except for the Yorkshire post-1998 subpopulation, more than 90% of the ancestors were known in other populations. But by the fourth generation, only Duroc is higher than 70%, Landrace has fallen below 70%, and the value is less than 50% for the Yorkshire breed. In summary, the completeness of the Duroc pedigree is better than that of the other two breeds. The requirements for pedigree integrity after the occurrence of FMD are also significantly higher than they were before the occurrence of FMD.

### Generation Interval

Computed average GI and divided into four selection paths in all breeds are shown in Table 2. As mentioned above, the average GI of all the breeds is over 2 years, so the breeding stocks born in the most recent 3 years of each subpopulation are set as the reference population. The results indicate that the GI of the population before the occurrence of FMD is shorter than that after the occurrence of FMD in all breeds. This might be due to the reduction of the scale of production, which reduced the demand for breeding stocks, and lead to the breeding age of breeding stocks is older. The lowest value of average GI was observed for Duroc breed (2.17 and 2.43 years) in pre-1998 and post-1998 subpopulation, respectively. In contrast, the longest GI are all obtained Landrace breed (2.30 and 2.63 years) in different period. When comparing the different selection paths within each breed in pre-1998 subpopulation, we can observe that the longest GI was the sire to son path and the shortest GI was found for the dam to son path in Duroc. On the other hand, there are the longest GI for the dam to daughter path and the shortest path for the sire to daughter path of the two maternal line breed. However, the longest path was all detected for the sire to son path in each breed in post-1998 subpopulation. The highest difference among selection paths within one breed was observed in Landrace in different subpopulation (0.26 and 0.38, respectively). The most balanced GI was detected for Duroc and Yorkshire (0.07 in pre-1998 subpopulation and 0.26 in post-1998 subpopulation) when comparing the individual paths of selection.

**Table 2.** Average generation intervals in evaluated breeds.

Breed	Duroc		Landrace		Yorkshire	
subpopulation	Pre-1998	Post-1998	Pre-1998	Post-1998	Pre-1998	Post-1998
Average generation interval	2.17	2.43	2.30	2.63	2.28	2.60
GI for sire–son path	2.20	2.66	2.20	2.96	2.21	2.78
GI for sire–daughter path	2.19	2.43	2.17	2.58	2.19	2.56
GI for dam–son path	2.13	2.33	2.31	2.62	2.34	2.52
GI for dam–daughter path	2.16	2.41	2.43	2.64	2.38	2.61

GI, generation interval.

### The Parameters for Genetic Variability Evaluation

#### Inbreeding and effective population size 

The summary statistics for each breed are listed in Table 1. The results showed that the proportion of inbred animals of all breeds has increased over time. In the Duroc breed, the proportion of inbred animals is significantly higher than in the other two breeds. However, although the proportion of inbreeding in the analyzed breeds is high the value of the mean inbreeding coefficient (Mean *F*) is moderate, ranging from 4.24% (Duroc) to 1.37% (Landrace) in the pre-1998 subpopulation and from 3.62% (Duroc) to 1.76% (Landrace) in the post-1998 subpopulation. With the occurrence of FMD, the average increase of inbreeding (∆*F*) of each breed presented a different trend. The Duroc breed was reduced, and the Landrace breed remained almost the same. However, ∆*F* showed a dramatic increase in the Yorkshire breed (from 0.58% to 1%). This result can also be seen in the effective population size (*N*_*e*_). Among the subpopulations pre- and post-1998, the Yorkshire post-1998 subpopulation had the lowest *N*_*e*_ (50 animals), while the Landrace had the highest *N*_*e*_ in both subpopulations.

#### The probability of gene origin.

Table 3 summarizes all of the calculated parameters for the gene origin analysis of the reference population. Among the pre-1998 subpopulation, the highest number of founders were detected (*f*) for Landrace (2,921), followed by Yorkshire (1,494), and Duroc (1,378). However, in the post-1998 subpopulation, the Landrace breed still had the highest number of founders, but the number for the Yorkshire breed was reduced to fewer than the Duroc breed. A similar trend also appeared for the effective number of founders (*f*_*e*_) and the equivalent number of founders’ genomes (*f*_*ge*_).

**Table 3.** Parameters derived from the probability of gene origin in the different reference populations in each breed.

Breed	Duroc		Landrace		Yorkshire	
subpopulation	Pre-1998	Post-1998	Pre-1998	Post-1998	Pre-1998	Post-1998
Total number of founders, *f*	1378	1049	2921	1393	1494	515
Effective number of founders, *f*_*e*_	174	167	457	169	369	80
Founder genome equivalent, *f*_*ge*_	65	36	337	71	192	36
*f*_*e*_/*f* ratio	0.13	0.16	0.16	0.12	0.25	0.16
*f*_*ge*_/*f*_*e*_ ratio	0.37	0.22	0.74	0.42	0.52	0.45
Number of ancestors to explain:						
50% of gene pool	56	35	150	42	105	22
75% of gene pool	169	103	415	133	295	74
100% of gene pool	1143	680	2598	1042	1255	350
GD	0.992	0.986	0.999	0.993	0.997	0.986
1 − GD (GD loss)	0.008	0.014	0.001	0.007	0.003	0.014
Proportion of unequal contributions of the founders in GD loss	37.36	21.56	73.74	42.01	52.03	45.00
Proportion of random genetic drift in GD loss	62.64	78.44	26.26	57.99	47.97	55.00

GD, genetic diversity; The equation of GD calculation is $GD=1-\frac{1}{2f_{ge}}$.

**Figure 3.**
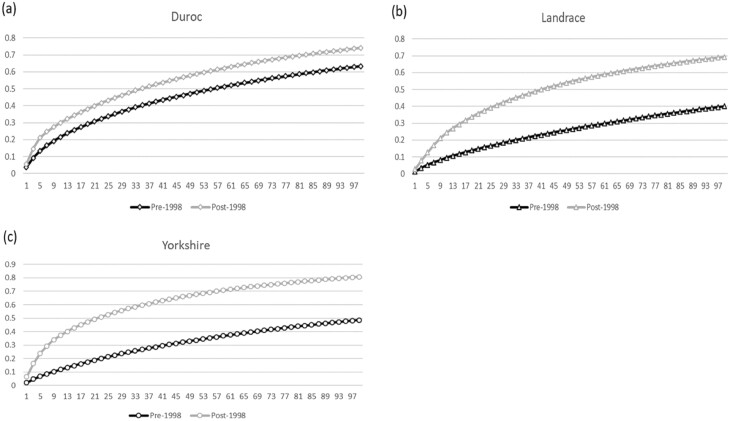
Accumulated marginal contribution of 100 major ancestors in the Taiwan swine breeds. (a) Duroc breed; (b) Landrace breed; (c) Yorkshire breed.

The highest values of *f*_*e*_ and *f*_*ge*_ were reached in the subpopulation of the Landrace breed before 1998 (457 and 337) and the subpopulation after 1998 (169 and 71). The lowest effective number of founders and founder genome equivalents were found in the Duroc and Yorkshire breeds (174 and 65 founders; 80 and 36 founders, respectively) in different subpopulations. After the occurrence of FMD, the *f*, *f*_*e*_, and *f*_*ge*_ of all breeds decreased. In comparing different subpopulations, the ratio of *f*_*e*_/*f* shows a similar trend in the Landrace and Yorkshire breeds; however, it increased in Duroc after the FMD outbreak. The highest value occurred in the Yorkshire breed (0.25) and the lowest value in Duroc (0.13) in the pre-1998 subpopulation. In the post-1998 subpopulation, Duroc and Yorkshire breeds had equal values (0.16), while the Landrace had the lowest value (0.12). The ratio of *f*_*ge*_/*f*_*e*_ decreased in all the breeds in this study over time. The lowest values calculated were for the Duroc breed in different subpopulations (0.37 in pre-subpopulation and 0.22 in post-subpopulation, respectively). The accumulated marginal contribution of 100 major ancestors is illustrated in Fig. 3a–c for the different breeds. Independent of breed, more ancestors are required to explain the gene origin in the pre-1998 subpopulation than in the post-1998 subpopulation. One hundred ancestors can only explain about 50% of the gene pool in pre-1998 subpopulations of the Landrace and Yorkshire breeds. However, only half this amount is needed for this purpose in the Duroc breed. In the post-1998 subpopulation, fewer than 50 ancestors are enough to explain 50% of the gene pool in all evaluated breeds. Comparing the breeds indicated that Landrace contains the largest amount of ancestors to explain its gene pool.

## DISCUSSION

From 1980 onward, the number of pigs and breeding farms increased because Taiwan began to export purebred herds to Southeast Asian countries, such as the Philippines and Thailand, and was a net exporter of prime pork meat to Japan, with the offal left for domestic consumption (Sung, 1993). However, the outbreak of FMD led to consequences such as the inability to export pigs and pork, the development of Taiwan’s purebred breeding pig industry stalled, and the population became closed. Therefore, this study uses pedigree information and different parameters (such as *F*, *N*_*e*_, *f*_*e*_ and *f*_*ge*_) to evaluate the GD of the population pre- and post-FMD to understand the impact on the GD of the sharp decline in the number of purebred stocks and subsequent population closure. The accuracy of parameters such as inbreeding coefficient, effective population size, and GD depends on the quality of the population pedigree. This study shows that the PCI of each breed can reach more than 60%, and the percentage of known ancestors in the fourth generation was higher than 70% in the Taiwan Duroc breed indicating a reasonable estimation of other pedigree parameters. Moreover, the correlated parameters of pedigree integrity of the subpopulation in the breeds in this study before and after the occurrence of FMD are very similar, so subsequent comparisons can be made. Furthermore, the pedigree quality of the Taiwanese pig population is moderate compared to some recent pedigree analyses of pigs. The completeness of our analyzed Duroc pedigrees was similar to that published by Melka and Schenkel in four Canadian swine breeds and more complete than the results reported by Tang et al. in three imported swine breeds in China (Melka and Schenkel, 2010; Tang et al., 2013) However, the Landrace and Yorkshire PCIs were worse than the previous studies. These differences in the quality of pedigree parameters between breeds found in our study might be caused by the dynamics and intensity of individual breeds used in commercial breeding programs. In addition, some differences may also be based on the impact of imports or the depth of pedigree knowledge of imported animals. These parameters ultimately affect the quality of the pedigree and thus the estimated inbreeding and GD.

In general, the generation intervals of males are slightly shorter than that of females (Melka and Schenkel, 2010). Similar results can be found in previous reports (Melka and Schenkel, 2010; Tang et al., 2013; Krupa et al., 2015). In this study, the pre-1998 subpopulation also showed the same trend, except for the Duroc breed. However, in the post-1998 subpopulation, each breed showed the opposite result, the generation interval of males is longer than females. The generation interval of both males and females increases over time. This may be because after the occurrence of FMD, Taiwan’s breeding pig industry shrank and could not be exported, demand for breeding stock has declined, so the mating strategy has shifted to the purebred population was centralized derived from certain animals, which leading to older breeding age of males and females in the herd. This is because breeders have no confidence in using new breeding animals, therefore would rather the current breeding stocks keep in production than allow new generation to join or even replace them. This is evident in the individual selection paths, especially in the sire to son path of each breed. This situation might be improved through on-farm testing, which has gradually matured in recent years in Taiwan. By performing performance testing, calculating estimated breeding values and establishing the selection index for various traits of purebred progeny, breeders can identify the best animals and mating them in younger age. It will helpful to shorten the generation distance and reduce breeding costs.

The observed values of the mean inbreeding coefficient over the breeds evaluated in this study were similar to the previous report. Krupa et al. (2015) have indicated a higher inbreeding coefficient for the Czech Duroc breed (approximately 3.6%), while for the Czech Landrace and Czech Large White breeds, it has not exceeded 3%. Furthermore, the inbreeding degree of Landrace in Taiwan was lower than the Czech Landrace. The rate of inbreeding (Δ*F*) is an effective criterion for measuring population health, and Nicholas has recommended a Δ*F* rate < 0.5%, whereas the Food and Agriculture Organization of the United Nations (FAO) has suggested a Δ*F* rate < 1% as a goal (Nicholas, 1989; FAO, 2000). All breeds meet the FAOs goal in the different subpopulations, but only the Landrace breed conforms to the rate indicated by Nicholas. The FAO suggested that *N*_*e*_ for a breed should be maintained above 50 in order to withstand the effects of inbreeding (FAO, 2000), while a size of 500 is essential to sustain the genetic variability and evolutionary potential of the population for several generations (Frankham et al., 2002). These authors defined at least 500 animals of *Ne* are needed to maintain GD in the population for several generations. From the perspective of current and past effective population sizes, efforts should be made for all the breeds analyzed in this study to enhance the *N*_*e*_ in order to construct a more varied population, although they are presently similar to the results of previously published literature, and all of them meet the minimum requirement for *N*_*e*_ (50 animals) (Melka and Schenkel., 2010; Tang et al., 2013; Krupa et al., 2015). As the biotechnology has advanced, it allow using genome information for the assessment and management of GD. Genomics data used for many alternative measures of inbreeding and genomic relationships. These measures are applied for managing GD in genome best contribution (GOC) selection schemes (Meuwissen et al., 2020). By using genomic information to measure different inbreeding coefficients and genomic relationships, such as drift or homozygosity-based inbreeding coefficients, it is possible to understand the true situation of allelic diversity (AD), and then choose the best GOC management plan. The similar results can be observed in previous study that different inbreeding coefficient value obtained by using genomic information and pedigree data (Grossi et al., 2017). These results all prove that the measurement derived only from the pedigree are not comprehensive enough, and many details in it need to be more clearly understood and applied through the use of genomic information. Therefore, genomic-based analyses should be performed in our future studies to elucidate the real causes of GD loss and to identify strategies for GD management. The *Ne* of Duroc and Landrace increased with time, but Yorkshire decreased with time, even more so in the post-1998 subpopulation. The *N*_*e*_ of Yorkshire breed has been reduced to a critical value (50 animals), which means that the Δ*F* rate of the current population has reached 1%. Though the Duroc breed still has the highest mean inbreeding coefficient, the Yorkshire breed has shortened the distance. According to these results, the Yorkshire breeders have recently switched to the strategy of constructing the population by import instead of breeding native breed (the percentage of unknown parents in the registered Yorkshire population is equal to 13%) and centralizing by using this imported livestock to multiply the nucleus herd. The newly introduced breeding stocks are often mated with their progeny, causing their Δ*F* rate to increase much faster than the other two breeds so that the usage of the Yorkshire breed in Taiwan is very limited. However, a previous study demonstrated that the reproductive performance of Yorkshire × Landrace (two-way crossbred) sows was better than that of purebred Landrace sows when both were mated with Duroc sires (Huang Y-H et al., 2002). This illustrated the advantage to incorporate the Yorkshire breed into the production system.

The assessment of GD is especially important in highly specialized livestock breeds because assisted reproduction techniques, such as artificial insemination and embryo transfer technologies, can potentially rapidly reduce the GD of a population (Vasconcellos et al., 2003). Parameters derived from the probability of gene origin analysis indicated an increased trend of GD loss with time in all three breeds in this study. A relatively small number of major ancestors are needed to explain the gene origin appears in the post-1998 subpopulation, which shows that the occurrence of FMD led to the decrease of GD. In general, the number of ancestors explaining the entire gene pool is still much greater than the results presented in the previous literature, showing that the present Taiwanese purebred stocks maintain a fairly good GD (Melka and Schenkel., 2010; Tang et al., 2013; Krupa et al., 2015).

To further explore the causes of GD loss, the *f*_*e*_/*f* ratio represents the reduction of GD due to the unequal contribution of founders. The *f*_*ge*_/*f*_*e*_ ratio can be used to quantify only the influence of genetic drift on the amount of GD. In general, if all the founders were to contribute equally, the total number of founders would be the same as *f*_*e*_. However, the *f*_*e*_ is usually lower than *f*, indicating there are unequal contributions of founders due to selection, namely that the breeders have preferentially chosen certain animals as parents (Melka and Schenkel, 2010). The lower these two ratios are, the strong the effect of unequal contributions of founders or random genetic drift is. The results of the probability of gene origin in the reference populations show the *f*_*e*_/*f* ratio was similar in these three breeds at different periods, which means they may be under the same degree of selection intensity in Taiwan. Over the past years, knowledge of the production system has allowed the adjustment of the selection intensity for growth and carcass traits (such as average daily gain and back fat) and reproductive traits (such as the longevity and litter size) in these three breeds. However, the Duroc breed presents the lowest *f*_*ge*_/*f*_*e*_ ratio in both subpopulations, indicating the significant effect of random genetic drift. Compared to previous studies of Canadian and Czech populations, the influence of random genetic drift in Taiwan is milder (Melka and Schenkel, 2010; Krupa et al., 2015). In this study, the impact of random genetic drift was substantial for all breeds. The highest value of overall GD lost was observed for the Duroc and Yorkshire breeds in the post-1998 subpopulation. This situation appears to have occurred because the previous results indicated in the Czech population were similarly due to the number of animals, along with the proportion of imported animals, was reduced during the period of analysis (Krupa et al., 2015).

Before the occurrence of FMD, Duroc GD loss was mainly due to random genetic drift, while Landrace and Yorkshire GD losses were due to the unequal contributions of founders. However, after the occurrence of FMD, the main cause of GD loss of the three breeds was random genetic drift. Although Duroc still has the highest degree of influence of random genetic drift (78.44%), the influence of random genetic drift in Landrace has been significantly increased (from 26.26% to 57.99%), which indicates that the dramatic decline of this population has had a greater impact than other two breeds on its GD. The main reason for such a high proportion of GD loss caused by random genetic drift (and other reasons such as the bottleneck effect) is the population shrinkage and the declining proportion of introduced animals in Taiwan after the outbreak of FMD. Our study compared the different subpopulations, which contrasted the historical and current status of genetic variability to clarify the impact of FMD. Previous studies had indicated that GD loss due to random genetic drift led to an increase in homozygosity and fixation of alleles (Fernández et al., 2005; Toro et al., 2006). The loss of diversity can be caused by random genetic drift or unequal contributions of the founders. This study confirmed that the selection of breeding stocks for specific traits by breeders has a certain impact on the loss of population GD in Taiwan. However, after the outbreak of FMD in Taiwan, the population size suddenly decline and the proportion of imported animals decreased, resulting in purebred populations mainly derived from breeding stocks with similar genetic backgrounds, which lead to an increase in the degree of random genetic drift. Therefore, to solve such problems, breeding strategies should be adjusted, such as introducing new genetic material and reducing inbreeding (Haile-Mariam et al., 2007) to reduce the effects of drift (Lacy, 1987) to further improve the level of GD. After long-term efforts, Taiwan finally was removed from the FMD epidemic area in June 2020, which provided another opportunities for pork exports. In the future, Taiwan breeders should refer to the results of previous and this study to formulate introduction and selection/breeding strategies to effectively reduce the impact of random genetic drift, and increase the GD of the entire purebred stocks, for providing a stable supply basis in the export market.

## CONCLUSION

In summary, pedigree quality of Duroc breed is better than the other breeds which might be related to that Duroc is used as sire and the influence exceed the other two maternal breeds. Therefore, for the Landrace and Yorkshire breeds, breeders in Taiwan need to record their pedigree more carefully to obtain more accurate evaluation results. The results of GI analysis indicated that Taiwanese breeders should adjust their management that mating the new generation in younger age according to the result of progeny test to shorten the generation interval. Genetic variability measurements show that after the FMD outbreak, random genetic drift played an important role in the loss of GD in Taiwanese swine breeds. Breeders should reduce the inbreeding rate, introduce genetically unrelated individuals and formulate better mating strategy (such as manage the breeding stock mating in younger breeding age) to increase the effective population size. The Δ*F* rate of the Yorkshire breeds has increased significantly. Therefore, in addition to introduction, breeders should pay more attention to the adjustment of breeding operation methods for Yorkshire breeds, with the goal of develop a Taiwanese Yorkshire population that is adapted to Taiwan’s environment, cautiously use foreign breeding stocks in the production system. Maximize the effect of foreign breeding stock, which thereby reducing the rate of Δ*F* and increasing GD in population.
